# Predictive performance of the Zulfiqar Frailty Scale for 12-month morbidity and mortality in community-dwelling older adults attending general practice

**DOI:** 10.25122/jml-2025-0094

**Published:** 2025-09

**Authors:** Abrar-Ahmad Zulfiqar

**Affiliations:** 1 Internal Medicine Department, Strasbourg University Hospitals, Strasbourg, France

**Keywords:** frailty syndrome, Zulfiqar Frailty Scale (ZFS), adverse events, falls, hospitalizations

## Abstract

Frailty in older adults is a syndrome associated with increased morbidity and mortality. The Zulfiqar Frailty Scale (ZFS) was developed to facilitate the assessment of frailty in general practice. This study aimed to assess the predictive capacity of the ZFS over 12 months for events such as falls, hospitalizations, changes in treatment, and mortality. A prospective study was conducted in a general practice over a 12-month period. Patients aged 65 and over were included and assessed using the ZFS at T0 months, followed by assessment at T12 months. Data collected included demographic information, medical history, hemoglobin level over the last 3 months, and hemoglobin level at 12 months, as well as the occurrence of adverse events (falls, fractures, hospitalizations, and death). Statistical analyses were performed using ROC curves to determine the performance of the ZFS scale. Of the 135 patients included, 24% were considered frail at T0, and this figure rose to 28% at T12 months. The ZFS showed good predictive capacity for the occurrence of falls (AUC = 0.75) and hospitalizations (AUC = 0.64). Frail patients, according to the ZFS, had a significantly higher risk of falling (*P* < 0.05) and were hospitalized more frequently (*P* < 0.001) than non-frail patients. Concerning mortality, although the number of deaths was low (3 deaths), the ZFS showed an AUC of 0.87, indicating a good predictive capacity. On the other hand, the prediction of fractures (AUC = 0.62) and new comorbidities (AUC = 0.51) performed less well. The ZFS is a promising tool for screening for frailty and predicting certain clinical events such as falls and hospitalizations. However, for more comprehensive predictions (fractures, comorbidities), association with other assessment tools is recommended.

## Introduction

In France, by 2070, older adults are expected to represent 29% of the population. This aging phenomenon is not new; the number of older adults has doubled almost every 50 years since 1920. It is linked to the rise in life expectancy since the end of the 19^th^ century [1]. One of our society's major challenges is to preserve the functions and autonomy of older adults. Frailty is a clinical syndrome that precedes the onset of dependency [2,3]. This condition is closely linked to increased morbidity and mortality, underlining the importance of early and accurate identification of frail people in primary care [4]. It is difficult to arrive at a consensus definition, as this notion is dynamic, evolving, and multidimensional, encompassing physical, physiological, biological, social, and environmental factors. Nevertheless, all authors agree that it is a major factor in morbidity and mortality [4,5]. Identifying frailty makes it possible to predict, over a period of 1 to 3 years, the risk of loss of autonomy, falls, institutionalization, hospitalization, and death [6]. In this context, the role of the general practitioner (GP) is essential. GP is the first healthcare professional in contact with the patient, able to suggest screening, manage follow-up, and coordinate any care that may be required. The gold standard for diagnosing and assessing frailty is the Comprehensive Geriatric Assessment (CGA) based on a multidimensional model. CGA is a multidimensional, structured approach used to assess the overall health of older adults. It explores medical, functional, cognitive, nutritional, social, and environmental areas to identify frailty and optimize care. The CGA thus enables the development of a personalized care plan aimed at maintaining independence and preventing dependency [7].

However, conducting a CGA is a time-consuming process that requires proficient geriatric skills. This highlights the need for a screening tool that is simple to use in primary care.

**Figure 1:**
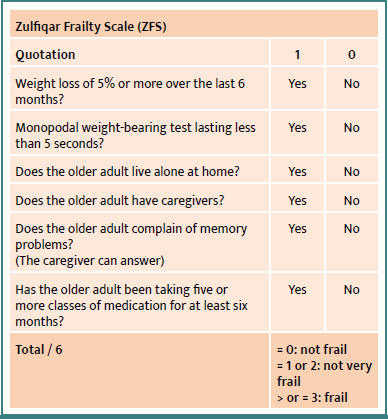
Zulfiqar Frailty Scale (ZFS)

To address this need, several frailty screening tools have been developed; however, no consensus has been reached on a universally reliable tool [6]. The Fried Phenotypic Frailty Scale, developed in the 1990s by teams led by Professor Linda Fried, an American epidemiologist and geriatrician, focuses on the concept of sarcopenia in the context of phenotypic frailty. However, this scale is difficult to use in ambulatory medicine due to its spatial constraints (requiring a 4.57 m walk), time requirements, and material needs, such as the use of a dynamometer [8]. The Zulfiqar Frailty Scale (ZFS) has been proposed to facilitate the identification of frailty during general medical consultations. It has been the subject of several published studies and has been tested and validated in a number of general practices. The results of the proof-of-concept study were highly satisfactory and reproducible, with similar findings observed in subsequent trials [9-13]. This quick and easy-to-use scale is based on six criteria (Figure 1), which, in the literature, are associated with a poor prognosis in terms of morbidity and mortality [14-17]. However, although frailty is a recognized predictor of morbidity and mortality, the predictive performance of the ZFS scale in this area remains to be thoroughly evaluated.

Each positive response ("yes") is scored 1 out of 6. The patient is considered "not frail" if the score is 0/6, "not very frail" if the score is 1 or 2/6, and "frail" if the score is 3/6 or greater.

The primary objective of our study was to assess the performance of ZFS in predicting the 12-month occurrence of adverse events, including falls, fractures, hospitalization, changes in treatment, institutionalization, dependence, and mortality.

Anemia is increasingly recognized as a potential biological marker of frailty in the elderly. It is associated with reduced muscle strength, functional decline, and an increased risk of morbidity and mortality. Although not part of the ZFS items, we chose to assess its association with frailty to explore whether hematological status could be linked to the ZFS classification and enhance its predictive capacity.

## Material and Methods

To answer our research question, a prospective study was set up. It was conducted in a general practice in Saint-Dizier, Haute-Marne, over a total period of 12 months. It began on April 20, 2024, and lasted a total of 12 months.

### Objective

This study aimed to evaluate the effectiveness of the ZFS frailty screening tool in predicting, over 12 months, the occurrence of a pejorative event such as falls, fractures, changes in treatment, unscheduled hospitalizations (including emergency room visits), institutionalization, and mortality, in a population of older adults undergoing general medical consultation.

### Secondary objective

The secondary objective of the study was to assess the potential association between frailty, as measured by the ZFS, and anemia. Anemia is a common condition in older adults and is increasingly described in the literature as a contributor to frailty due to its impact on physical performance, fatigue, and reduced physiological reserve. By exploring this relationship, we aimed to determine whether the presence of anemia correlates with frailty status within the ZFS framework and whether anemia could complement this clinical scale as a biological indicator.

### Inclusion/exclusion criteria

Eligible patients were consecutively recruited during routine consultations in a general practice between April 2024 and April 2025. All patients meeting the inclusion criteria were informed about the study and invited to participate. Only those who provided signed informed consent were included in the cohort. Patients had to be aged 65 or older, living at home, consulting an outpatient GP, and have an ADL (Activities of Daily Living) score of four or higher. Patients under 65, living in nursing homes, with an ADL of less than four, and who had not signed the consent form, were not included.

### Data collected and analyzed

The ZFS score was calculated based on six indicators assessing the main functions of an elderly person, chosen according to their geriatric relevance as defined in the scientific literature [14-17]. It was carried out at T0 and T12 months. Data were collected during face-to-face consultations in the general practitioner’s office. The ZFS scale was administered by the general practitioner during the clinical visit, based on direct questioning of the patient and observation. Sociodemographic and clinical data (gender, weight, height, BMI, hemoglobin levels, comorbidities), ADL calculation, medical and surgical history, number of usual treatments, hemoglobin level for the last three months prior to collection at T0 months, medical events occurring after the first assessment at T12 months (fall, fracture, addition or modification of treatments, unscheduled hospitalization, institutionalization and occurrence of death) as well as collection of hemoglobin level at 12 months, were extracted from patients’ medical records and confirmed with the patients. All data were recorded on standardized paper forms and then entered into a dedicated Excel spreadsheet for analysis. Anemia was defined as a hemoglobin level below 12 g/dL for women and 13 g/dL for men.

### Statistical analysis

Statistics were compiled using RStudio software version 2023.09.0. A descriptive analysis was carried out, with qualitative variables expressed as numbers (percentages) and quantitative variables as mean and standard deviation. ROC curves were generated to illustrate and evaluate the performance of the ZFS scale, and to determine its optimal threshold values. The ROC curve is the result of a prediction model that uses one of the two variables to predict the other. To determine whether a classifier is good, we need to obtain a curve above the dotted line, which gives the results for a random dispersion. The closer the curve is to the top-left corner, the better the prediction. The area under the curve (AUC) quantifies this. An AUC close to 1 indicates perfect association of the "Frail" modality with the "Yes" response and the "Non-frail" modality with the "No" response. The closer the AUC is to 1, the more "obvious" the association of the modalities. The Fisher test was used when one of the crosses involved fewer than five individuals. In cases where all crosses involved at least five individuals, the chi-square test was used. For both tests, the *P* value was analyzed. If the *P* value was less than 0.05, then the modalities of the groups comprising the two cross-tabulated variables were significantly different.

## RESULTS

135 older adults were included and followed up, with no refusals noted, including 68 women. Complete results at T0 and T12 months are detailed in Tables 1 and 2.

**Table 1:** Description of the population at T0

Variables	Number (%)	Average (standard deviation)
**Gender**		
Woman	68 (50%)	/
Men	67 (50%)	/
**Number of usual treatments**	/	6.9 (3.3)
Missing	/	1
**ADL (/6)**	/	5.95 (0.27)
**Weight (kgs)**	/	77 (16)
**Size (cm)**	/	164 (11)
Missing	/	21
BMI	/	28.5 (5.4)
Missing	/	23
**Hemoglobin last 3 months (g/dl)**	/	14.01 (1.33)
**Presence of anemia (last 3 months)**		
Yes	5 (3.7%)	/
No	130 (96%)	/
**1^st^ question: weight loss >=5% of usual weight over 6 months**		
Yes	10 (7.4%)	/
No	125 (93%)	/
**2^nd^ question: Single-modal weight-bearing test < 5 seconds**		
Yes	46 (34%)	/
No	89 (66%)	/
**Question 3: Are there more than 5 therapeutic classes?**		
Yes	86 (64%)	/
No	49 (36%)	/
**Question 4: Does the older adult live alone at home?**		
Yes	44 (33%)	/
No	91 (67%)	/
**5^th^ question: presence of caregivers?**		
Yes	13 (9.6%)	/
No	122 (90%)	/
**6^th^ question: Any memory problems?**		
Yes	11 (8.1%)	/
No	124 (92%)	/
**ZFS score (/6)**	**/**	**1.56 (1.18)**

ADL, activity of daily living; BMI, body mass index; ZFS, Zulfiqar Frailty Scale

**Table 2:** Results at T12 months

Occurrence of a fall in the past 12 months		
Yes	15 (11%)	/
No	120 (89%)	/
**Hospitalization over the past 12 months**		
Yes	35 (26%)	/
No	100 (74%)	/
**Deaths over the past 12 months**		
Yes	3 (2.2%)	/
No	132 (98%)	/
**Nursing home admissions over the past 12 months**		
Yes	3 (2.2%)	/
No	132 (98%)	/
**Fractures over the past 12 months**		
Yes	14 (10%)	/
No	121 (90%)	/
**Adding/modifying treatments over the past 12 months**		
Yes	52 (39%)	/
No	83 (61%)	/
**Onset of comorbidity past 12 months**		
Yes	58 (43%)	/
No	77 (57%)	/
**ADL at 12 months**	/	6 (0)
Missing	/	1
**Hemoglobin at 12 months**	/	13.83 (1.37)
Missing	/	6
**Presence of anemia over the past 12 months**		
Yes	10 (7.8%)	/
No	119 (92%)	/
Missing	/	6
**Weight loss >=5% of usual weight over 12 months**		
Yes	20 (15%)	/
No	114 (85%)	/
Missing	/	1
**Does the older adult live alone at home? (T12 months)**		
Yes	42 (31%)	/
No	92 (69%)	/
Missing	/	1
**Presence of caregivers (T12 months)**		
Yes	15 (11%)	/
No	119 (89%)	/
Missing	/	1
**Presence of memory problems? (T12 months)**		
Yes	15 (11%)	/
No	119 (89%)	/
Missing	/	1
**More than 5 therapeutic classes (T12 months)**		
Yes	87 (65%)	/
No	47 (35%)	/
Missing	/	1
**Single-leg support test <5 seconds? (T12 months)**		
Yes	48 (36%)	/
No	85 (64%)	/
Missing	/	2
**New evaluation of the ZFS 12-month scale (ZFS)**	/	1.71 (1.23)
Missing	/	3
**ZFS score at T0**		
Frail	32 (24%)	/
No, and not very frail	103 (76%)	/
**ZFS score to T12**		
Frail	38 (28%)	/
No, and not very frail	97 (72%)	/

ADL, activity of daily living; BMI, body mass index; ZFS, Zulfiqar Frailty Scale

Given that the *P* value is less than 0.05, we can conclude that the two variables are significantly related, as the patient's ZFS class did not appear to vary significantly between T0 and T12 (Table 3).

**Table 3:** Contingency table between ZFS score at T0 and ZFS score at T12

		T0	
		Frail	No, and not very frail
**T12**	**Frail**	27	11
	**No, and not very frail**	5	92

Chi-square test: *P* value < 0.001

The *P* values were greater than 0.05 and therefore not significant. Having anemia or not did not differ significantly between frail and non-frail people (Tables 4 and 5).

**Table 4:** Correlation between anemia and frailty syndrome assessed using the ZFS scale

Anemia and ZFS score at T0				
	ZFS		*P* value	
	Frail, *n* = 32	Non-frail, *n* = 103		
**Anemia in the last 3 months**				
Yes	2 (6%)	3 (3%)	0,592	Fisher
No	30 (94%)	100 (97%)		
**Anemia at T12 months**				
Yes	3 (10%)	7 (7%)	0,693	Fisher
No	26 (90%)	93 (93%)		
Missing	3	3		

**Table 5:** Anemia and ZFS score at T12 months

Anemia and ZFS score at T12 months				
	ZFS		*P* value	
	Frail, *n* = 35	Non-frail, *n* = 97		
**Anemia in the last 3 months**				
Yes	3 (9%)	2 (2%)	0,116	Fisher
No	32 (91%)	95 (98%)		
**Anemia at T12 months**				
Yes	5 (15%)	5 (5%)	0,148	Chi2
No	28 (85%)	90 (95%)		
Missing	2	2

- Occurrence of a fall in the past 12 months:
  The chi-square test yielded a *P* value of less than 0.05, indicating a significant relationship between the modalities of the different variables at the 5% threshold. Having fallen at T12 months was therefore an indicator of potential frailty, and the variables were significantly related (Table 6).
  We can say that people who have fallen were more likely to be considered frail on the ZFS scale than people who have not fallen.
- Hospitalization over the past 12 months:
  The chi-square test yielded a *P* value of less than 0.05, indicating a significant relationship between the modalities of the different variables at the 5% threshold. It can be said that, on average, the proportion of people hospitalized was significantly higher among the frail than among the non-frail (Table 6). Having been hospitalized in the last 12 months was therefore an indicator of frailty, and the variables were significantly related.
- Occurrence of a fall in the past 12 months:
  The Fisher test had a *P* value well below 0.05, indicating a significant relationship between the modalities of the different variables at the 5% threshold. Having had a fall in the last 12 months was therefore an indicator of potential frailty, and the variables were significantly related (Table 7).
  We can say that people who have had a fall in the last 12 months were more likely to be considered frail on the ZFS scale than people who have not had a fall.
- Hospitalization over the past 12 months:
  The chi-square test yielded a *P* value well below 0.05, indicating a significant relationship between the modalities of the different variables at the 5% threshold. It can be said that, on average, the proportion of people hospitalized in the last 12 months was significantly higher among the frail than among the non-frail (Table 7).
  Having been hospitalized in the last 12 months was therefore an indicator of frailty, and the variables were significantly related.
- Deaths over the past 12 months:
  The Fisher test had a *P-*value well below 0.05, indicating a significant relationship between the modalities of the different variables at the 5% threshold.
  It can be said that, on average, the proportion of people who died was significantly higher in the frail than in the non-frail (Table 7).

**Table 6:** Comparison of ZFS scores at T0 according to patient frailty

Zulfiqar Frailty Scale at t0			*P* value						
	Frail, *n* = 32	Non-frail, *n* = 103	Fisher	Chi-square	Significance	Se	Sp	PPV	NPV
**Occurrence of a fall in the past 12 months**									
Yes	8 (25%)	7 (7%)		0,0111	*	25%	93%	53%	80%
No	24 (75%)	96 (93%)							
**Fractures over the past 12 months**									
Yes	3 (9%)	11 (11%)	1,0000		n.s.	9%	89%	21%	76%
No	29 (91%)	92 (89%)							
**Hospitalization over the past 12 months**									
Yes	14 (44%)	21 (20%)		0,0163	*	44%	80%	40%	82%
No	18 (56%)	82 (80%)							
**Onset of comorbidity 12 months**									
Yes	13 (41%)	45 (44%)		0,9192	n.s.	41%	56%	22%	75%
No	19 (59%)	58 (56%)							
**Add/modify treatments 12 months**									
Yes	14 (44%)	38 (37%)		0,6254	n.s.	44%	63%	27%	78%
No	18 (56%)	65 (63%)							
**Anemia at 12 months**									
Yes	3 (10%)	7 (7%)	0,6928		n.s.	10%	93%	30%	78%
No	26 (90%)	93 (93%)							
**Deaths over the past 12 months**									
Yes	2 (6%)	1 (1%)	0,1398		n.s.	6%	99%	67%	77%
No	30 (94%)	102 (99%)							

**** < 0.0001 *** < 0.001 ** < 0.01 * < 0.05 Se, sensibility; Sp, specificity; PPV, positive predictive value; NPV, negative predictive value. n.s., Nonsignificant

**Table 7:** Comparison of ZFS scores at T12 according to patient frailty

Zulfiqar Frailty Scale (ZFS) at T12			*P*-value						
	Frail, *n* = 32	Non-frail, *n* = 103	Fisher	Chi2	Significance	Se	Sp	PPV	NPV
**Occurrence of a fall in the past 12 months**									
Yes	11 (29%)	4 (4%)	0,0001		***	29%	96%	73%	78%
No	27 (71%)	93 (96%)							
**Fractures over the past 12 months**									
Yes	7 (18%)	7 (7%)		0,1082	n.s.	18%	93%	50%	74%
No	31 (82%)	90 (93%)							
**Hospitalization over the past 12 months**									
Yes	17 (45%)	18 (19%)		0,0037	**	45%	81%	49%	79%
No	21 (55%)	79 (81%)							
**Onset of comorbidity 12 months**									
Yes	17 (45%)	41 (42%)		0,9463	n.s.	45%	58%	29%	73%
No	21 (55%)	56 (58%)							
**Add/change treatment 12 months**									
Yes	18 (47%)	34 (35%)		0,2602	n.s.	47%	65%	35%	76%
No	20 (53%)	63 (65%)							
**Anemia 12 months**									
Yes	5 (15%)	5 (5%)		0,1635	n.s.	15%	95%	50%	76%
No	29 (85%)	90 (95%)							
**Deaths over the past 12 months**									
Yes	3 (8%)	0 (0%)	0,0210		*	8%	100%	100%	73%
No	35 (92%)	97 (100%)							

**** < 0.0001 *** < 0.001 ** < 0.01 * < 0.05 Se, sensibility; Sp, specificity; PPV, positive predictive value; NPV, negative predictive value. n.s., non-significant

### ROC curves

**Figure 2:**
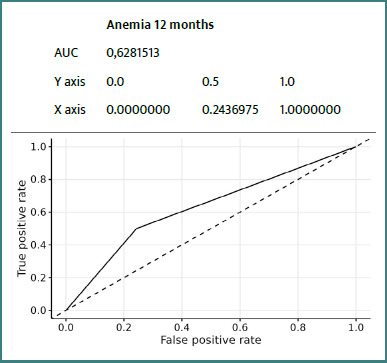
ROC Anemia T12 Months ZFS T12 Months

**Figure 3:**
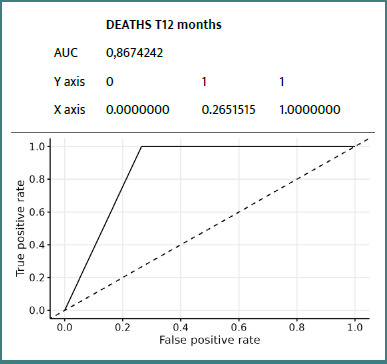
ROC Death T12 Months ZFS T12 Months

**Figure 4:**
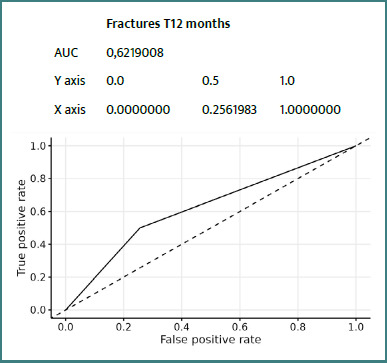
ROC Fracture T12 Months ZFS T12 Months

**Figure 5:**
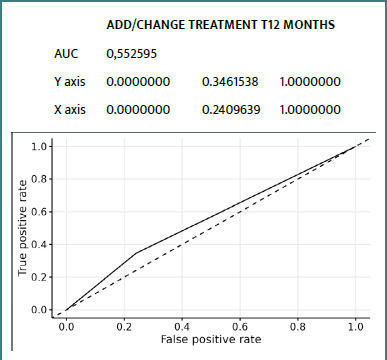
ROC Add/Change Treatment T12 Months ZFS T12 Months

**Figure 6:**
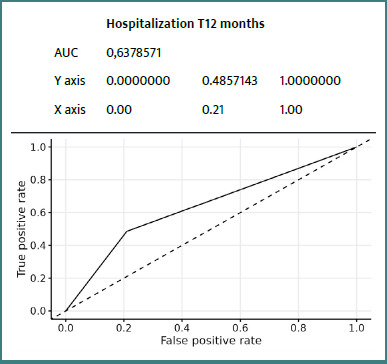
ROC Hospitalization T12 Months ZFS T12 Months

**Figure 7:**
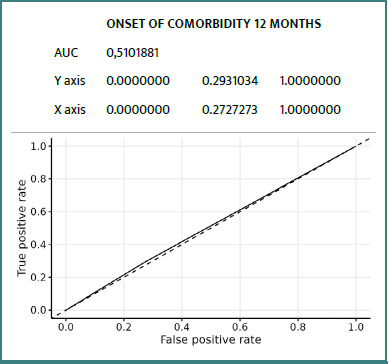
ROC Comorbidity Occurrence T12 Months ZFS T12 Months

**Figure 8:**
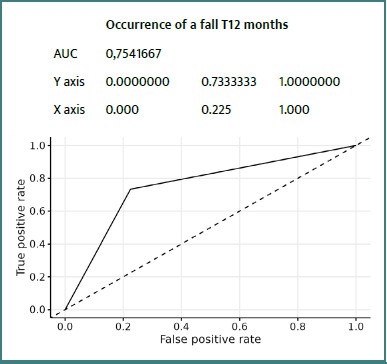
ROC Fall Occurrence T12 Months ZFS T12 Months

Details of ROC curves for the ZFS variable at 12 months:

We can see (Figures 2-8) that two variables had an AUC of 0.7 (Figures 3 and 8), indicating that these variables correctly predicted the frail or non-frail class.

## DISCUSSION

This prospective study confirms the value of the ZFS in general practice, particularly for predicting certain adverse events at 12 months in elderly patients. Integrated analysis of the results suggests that the ZFS is particularly effective in predicting falls, hospitalizations, and, to a lesser extent, mortality. The occurrence of falls was strongly correlated with frailty status as assessed by the ZFS, with an AUC of 0.75, indicating good discriminatory ability. This result was consistent with those of Kojima *et al*. [14] and Fried *et al*. [8], who demonstrated that frailty increases the risk of falls by decreasing muscle strength, postural stability, and walking speed. Early identification of these subjects could justify targeted interventions (balance program, muscle strengthening) as recommended by Giné-Garriga *et al*. [18]. Regarding hospitalizations, the ZFS demonstrated a moderate but significant AUC (0.64), consistent with the observations of Gill *et al*. [19], who reported that frail individuals utilize more healthcare services related to exacerbations of chronic conditions and infections. The integration of the ZFS score into a predictive model in primary care could therefore help to anticipate these uses and plan personalized follow-up. The ZFS score also demonstrated a high predictive ability for mortality (AUC = 0.87), although this data should be interpreted with caution due to the low number of deaths (*n* = 3). This result is consistent with the findings of Studenski *et al*. [20], who identified frailty as an independent factor in death, reinforcing the value of early screening.

However, the prediction of fractures and comorbidities was more limited (AUC of 0.62 and 0.51, respectively). This can be explained by the absence of bone parameters (bone mineral density, history of fractures) or biological parameters in the ZFS, as highlighted by Kanis *et al*. [21] with the FRAX tool. Similarly, the occurrence of new comorbidities depends on many contextual factors that are difficult to capture using a primarily clinical scale.

With regard to treatment adjustments and the occurrence of new comorbidities, the AUC of around 0.55 reflects poor predictive performance. These results highlight the limitations of the ZFS scale in capturing the complexity of drug interactions and the progression of comorbidities in older patients [22]. The addition of biological or cognitive criteria, as suggested by Clegg *et al*. [16], could improve this prediction. The link between anemia and frailty, although often reported [5,23,24], has not been confirmed in this cohort. The absence of a significant difference between anemic and non-anemic patients, regardless of the measurement time, may be attributed to a low prevalence of anemia (3.7% at T0), which limits statistical power. However, several studies, including those by Palmer *et al*. [23], show that anemia increases frailty through a decrease in functional reserve. The absence of a statistical link in our study does not, however, rule out its potential role, particularly in more severe populations or over longer periods of time. Longitudinal assessment between T0 and T12 shows stability of frailty for most patients, a result consistent with the work of Rockwood *et al*. [17], who emphasize that transitions between states of frailty are rare over short periods, except in the event of an acute event. This stability suggests that the ZFS may be useful not only for initial screening but also for follow-up over time.

Finally, methodological limitations must be taken into account, including a small sample size, a single-center design, a low number of rare events (such as death and institutionalization), and a lack of control for certain biases (subjective criteria, such as memory impairment). Nevertheless, the results provide a relevant insight into the use of ZFS in primary care practice.

### Prospects

The integration of the ZFS into the daily practice of general practitioners could facilitate the systematic identification of patients at risk and initiate appropriate early interventions. However, its effectiveness would be enhanced by a complementary approach based on a multidimensional geriatric assessment [7], or by the addition of biomarkers (e.g., hemoglobin, C-reactive protein) as suggested by Rockwood and Studenski [17,20].

## Conclusion

The Zulfiqar Frailty Scale proved to be a useful tool for screening for frailty in primary care, with good predictive capacity for certain clinical events, including falls, hospitalization, and death. However, its use for predicting other events (fractures, comorbidities) remains limited, suggesting the need to combine it with other tools for a more comprehensive assessment. These results underline the importance of early detection of frailty in order to optimize management and improve the quality of life for older patients. Its integration into clinical practice could improve the care of older adults; however, its combined use with other tools may be considered for a more comprehensive assessment of frailty.

## Data Availability

The datasets used and/or analyzed during the current study are available from the corresponding author upon request.
